# Doxorubicin induces an alarmin-like TLR4-dependent autocrine/paracrine action of Nucleophosmin in human cardiac mesenchymal progenitor cells

**DOI:** 10.1186/s12915-021-01058-5

**Published:** 2021-06-16

**Authors:** Sara Beji, Marco D’Agostino, Elisa Gambini, Sara Sileno, Alessandro Scopece, Maria Cristina Vinci, Giuseppina Milano, Guido Melillo, Monica Napolitano, Giulio Pompilio, Maurizio C. Capogrossi, Daniele Avitabile, Alessandra Magenta

**Affiliations:** 1 Experimental Immunology Laboratory, Istituto Dermopatico dell’Immacolata, IDI-IRCCS, Via Monti di Creta 104, 00167 Rome, Italy; 2 Unit of Vascular Biology and Regenerative Medicine, Centro Cardiologico Monzino, IRCCS, Via Carlo Parea 4, 20138 Milan, Italy; 3 Unit of Cardiology, IDI-IRCCS, Rome, Italy; 4 Clinical Epidemiology Unit, IDI-IRCCS, Rome, Italy; 5 Department of Biomedical, Surgical and Dental Sciences, University of Milan, Via Festa del Perdono 7, 20122 Milan, Italy; 6 Laboratory of Cardiovascular Science, National Institute on Aging (NIA), National Institutes of Health (NIH), 251 Bayview Blvd, Baltimore, MD 21224 USA; 7 Division of Cardiology, Johns Hopkins Bayview Medical Center, Baltimore, MD USA; 8 Idi Farmaceutici S.r.l., Via dei Castelli Romani 83/85, 00071 Pomezia (Rome), Italy; 9 National Research Council of Italy (CNR), Institute of Translational Pharmacology IFT, Via Fosso del Cavaliere 100, 00133 Rome, Italy

**Keywords:** Doxorubicin, Anthracyclines, Ultraviolet radiation, Alarmin, Cardiotoxicity

## Abstract

**Background:**

Doxorubicin (Dox) is an anti-cancer anthracycline drug that causes double-stranded DNA breaks. It is highly effective against several types of tumours; however, it also has adverse effects on regenerative populations of normal cells, such as human cardiac mesenchymal progenitor cells (hCmPCs), and its clinical use is limited by cardiotoxicity. Another known effect of Dox is nucleolar disruption, which triggers the ubiquitously expressed nucleolar phosphoprotein Nucleophosmin (NPM) to be released from the nucleolus into the cell, where it participates in the orchestration of cellular stress responses. NPM has also been observed in the extracellular space in response to different stress stimuli; however, the mechanism behind this and its functional implications are as yet largely unexplored. The aim of this study was to establish whether Dox could elicit NPM secretion in the extracellular space and to elucidate the mechanism of secretion and the effect of extracellular NPM on hCmPCs.

**Results:**

We found that following the double-strand break formation in hCmPCs caused by Dox, NPM was rapidly secreted in the extracellular space by an active mechanism, in the absence of either apoptosis or necrosis. Extracellular release of NPM was similarly seen in response to ultraviolet radiation (UV). Furthermore, we observed an increase of NPM levels in the plasma of Dox-treated mice; thus, NPM release also occurred in vivo. The treatment of hCmPCs with extracellular recombinant NPM induced a decrease of cell proliferation and a response mediated through the Toll-like receptor (TLR)4. We demonstrated that NPM binds to TLR4, and via TLR4, and nuclear factor kappa B (NFkB) activation/nuclear translocation, exerts proinflammatory functions by inducing IL-6 and COX-2 gene expression. Finally, we found that in hCmPCs, NPM secretion could be driven by an autophagy-dependent unconventional mechanism that requires TLR4, since TLR4 inhibition dramatically reduced Dox-induced secretion.

**Conclusions:**

We hypothesise that the extracellular release of NPM could be a general response to DNA damage since it can be elicited by either a chemical agent such as Dox or a physical genotoxic stressor such as UV radiation. Following genotoxic stress, NPM acts similarly to an alarmin in hCmPCs, being rapidly secreted and promoting cell cycle arrest and a TLR4/NFκB-dependent inflammatory response.

**Supplementary Information:**

The online version contains supplementary material available at 10.1186/s12915-021-01058-5.

## Background

Nucleophosmin (NPM) is a multifunctional phosphoprotein that shuttles between the nucleolus and cytoplasm [1, 2] facilitating the transport of proteins that possess nuclear localisation and export signals [3, 4].

Intracellular NPM has a role in the cell cycle, DNA damage repair [5], has nucleic acid-binding and chaperone activity [6] and regulates the stability and activity of transcription factors including p53 and nuclear factor kappa B (NFκB) [7, 8].

In human macrophages, it has been shown that lipopolysaccharides (LPS) treatment induced extracellular NPM release, which in turn activates inflammatory pathways, suggesting an alarmin-like function [9]. NPM has been shown to be actively secreted by lung cancer cells in response to serum starvation, to be associated with microRNAs and to protect them from degradation in the extracellular space [10].

Doxorubicin (Dox) is an anthracycline used as a chemotherapeutic agent in several types of tumours, whose use is limited by dose-dependent cardiotoxicity [11].

We previously showed that after Dox treatment, murine (m) cardiac progenitor cells (CPCs) and neonatal rat cardiomyocytes (NRMCs) underwent a rapid nucleolar stress [12]. The latter was characterised by NPM delocalisation from the nucleolus to the nucleoplasm, nucleolar disruption, new rRNA synthesis and ribosome biogenesis arrest, p53 stabilisation and transcriptional activation, that in turn, promoted apoptosis [12].

Nucleolar disruption represents an early response to stress and an important mechanism of Dox-induced cardiotoxicity [12]. The nucleolus, in fact, is a dynamic structure considered as a stress sensor [13]. Dox-induced cardiomyopathy is characterised by areas of interstitial fibrosis associated with apoptotic and necrotic cardiomyocytes [14]; further, cardiotoxicity also affects resident CPC [15].

Although the mechanisms leading to Dox-induced cardiotoxicity, have yet to be fully elucidated, oxidative stress and DNA damage are thought to play a role in such a process [11].

The aim of this study was to establish whether genotoxic stress induces NPM secretion and its role in cardiotoxicity.

## Results

### Dox induces a rapid release of NPM in the absence of oxidative stress, apoptosis and necrosis

Dox induces nucleolar stress, double-strand breaks (DSBs) and a rapid NPM delocalisation from the nucleolus to the nucleoplasm in mCPC and NRCMs [12]. Dox effect on NPM intracellular localisation, extracellular secretion and new rRNA synthesis in hCmPCs was analysed.

**Fig. 1:**
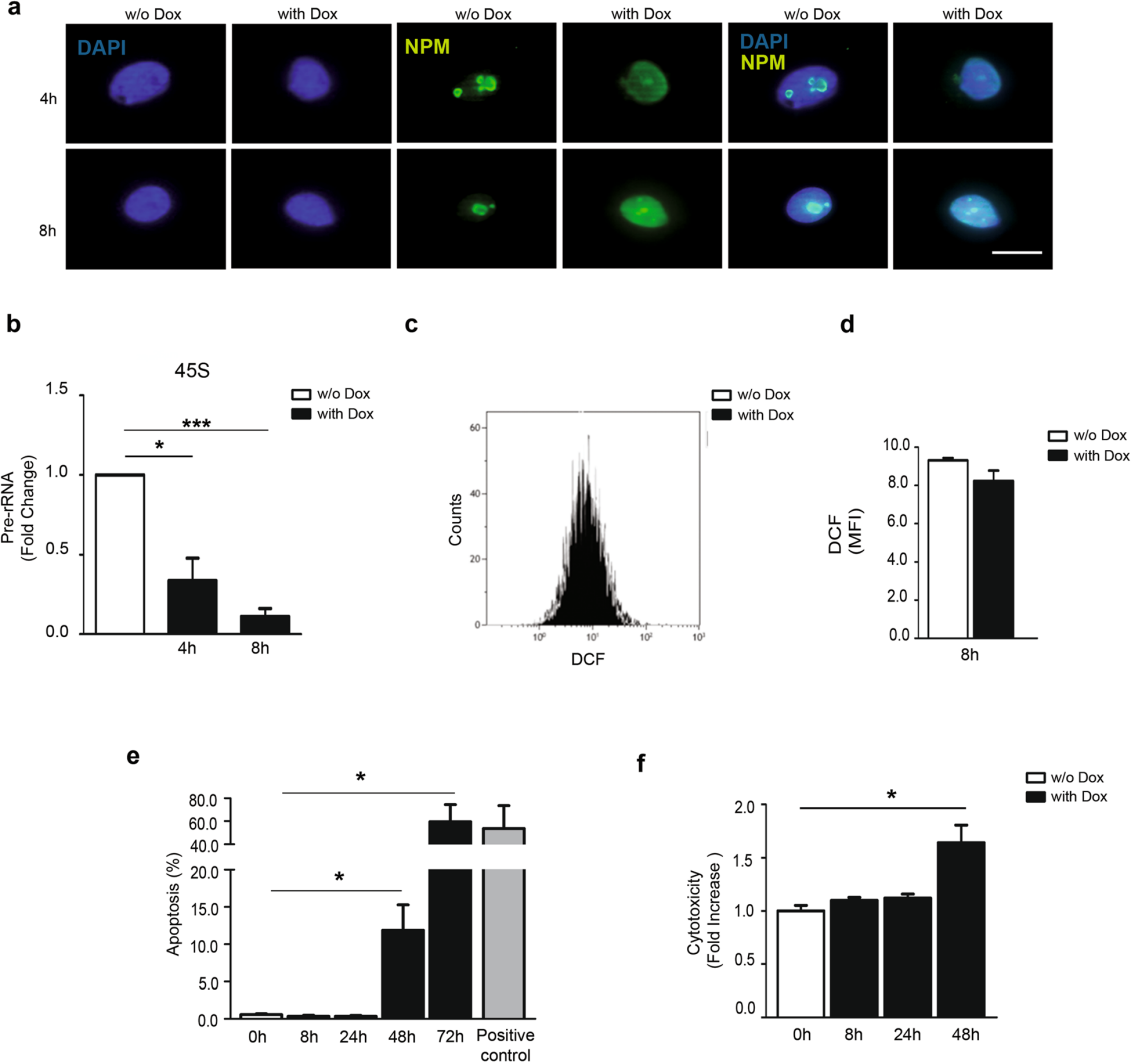
Dox induces nucleolar stress in hCmPCs in absence of ROS, apoptosis and cytotoxicity. **a** hCmPCs treated with 1 μM Dox for 4 h and 8 h (right upper and lower panels, respectively) or DMSO as control (w/o Dox; left upper and lower panels). Representative images of immunofluorescence of NPM (green) and nuclei counterstained with DAPI (blue). NPM is localised within nucleoli in control-treated cells and delocalises into the nucleoplasm upon Dox treatment. Scale bar 10μm. **b** Dox inhibits new rRNA synthesis in hCmPCs. Pre-rRNA 45S levels were measured by qRT-PCR and normalised to 18S expression (*n* = 4 for each group *P < 0.05; ***P < 0.001 vs Ctrl w/o Dox t=0h). **c** Representative image of DCF FACS measurements of hCmPCs treated or not with 1μM Dox for 8h. Serum starved hCmPCs were treated with 1 μM Dox for 8h. After treatment, cells were treated with DCF for ROS detection. **d** Bar graph representing mean fluorescence intensity (MFI) of DCF. Dox treatment does not significantly modulate ROS compared to untreated cells (*n* = 4 for each group). **e** Representative image of Annexin V FACS measurements of hCmPCs treated or not with 1μM Dox for 8, 24, 48, and 72h. After treatment, cells were stained with Annexin V for apoptosis detection. 30 min of 10mM H_2_O_2_ was used as positive control. Dox treatment significantly induced apoptosis at 48h and 72h compared Ctrl cells (*n* = 4 for each group; *P < 0.05 vs t=0h). **f** Representative image of cytotoxicity in Dox-treated hCmPCs. hCmPCs were treated with 1 μM Dox and analysed for cytotoxicity at different time points described in the figure. hCmPCs exhibited a significant cell toxicity only at 48h of treatment with Dox (*n* = 6; *P < 0.05)

hCmPCs treated for 4 or 8h with Dox showed a nucleolar delocalisation of NPM, that appeared dispersed in the nucleoplasm (Fig. 1a). To assess the nucleolar disruption and nucleolar stress, we measured the transcription rate of precursor rRNA45S normalised to the mature rRNA18S, as previously described [12]. Pre-rRNA synthesis was significantly decreased at both 4 and 8h following Dox (Fig. 1b).

In addition, hCmPCs apoptosis and ROS levels were measured 8h after Dox exposure. At this time-point, upon treatment with 1μM Dox, we did not detect significant increase of ROS (Fig. 1c, d), apoptosis (Fig. 1e, Additional file 1: Figure S1a), and cytotoxicity (Fig. 1f). In keeping with our previous published data, we observed apoptosis and cytotoxicity only after 48h of Dox exposure in hCmPCs (Fig. 1e, f) [16]. These findings confirm that nucleolar stress is an early response to stress preceding any induction of both ROS formation and apoptosis in hCmPCs, as shown in murine CPCs [12].

**Fig. 2:**
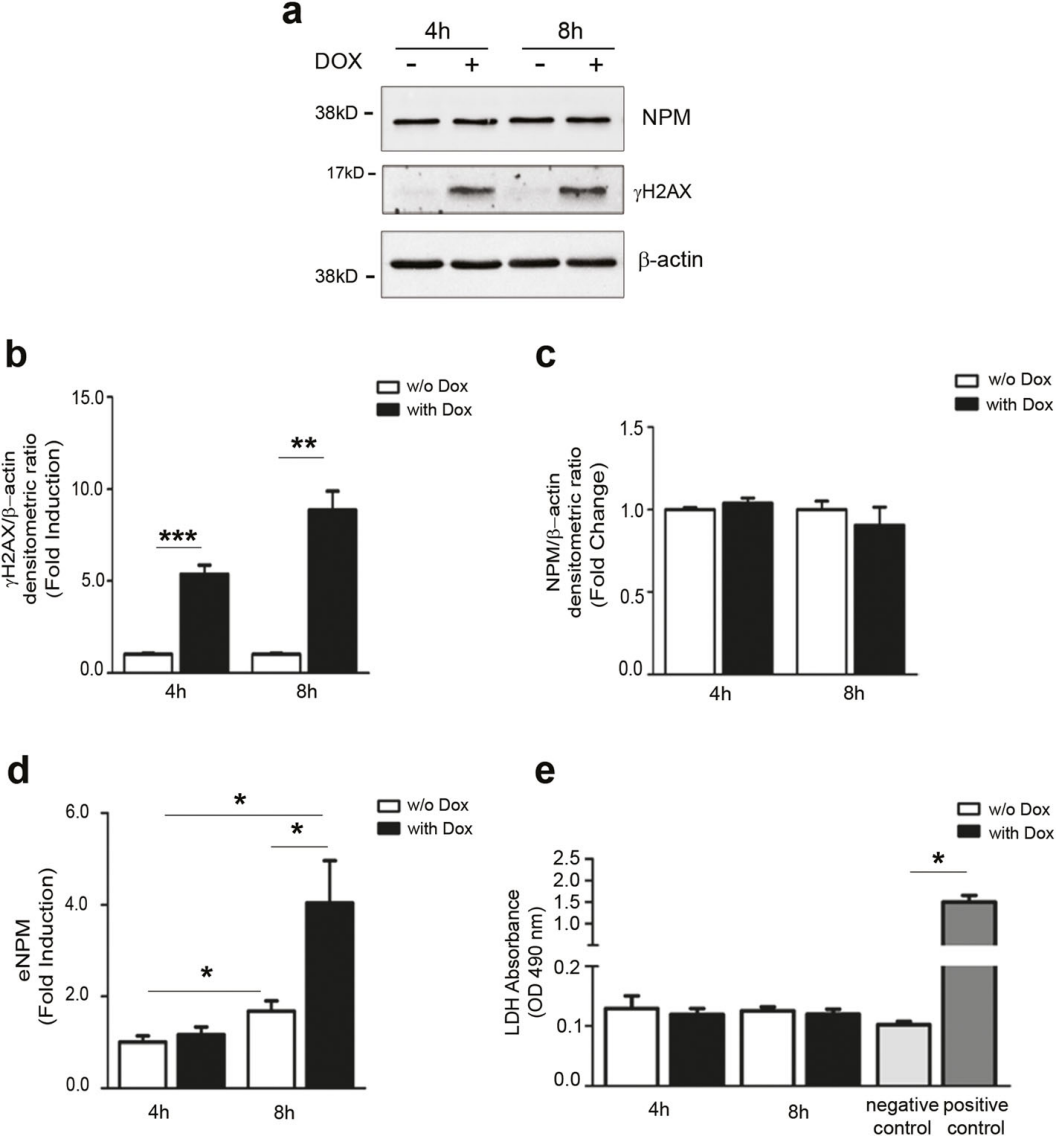
Dox-induced DSBs elicit a rapid NPM secretion in the absence of necrosis. **a** Representative WB using γH2AX and NPM antibodies showed that γH2AX levels were induced upon 1μM Dox in hCmPCs after 4 and 8h of treatment while NPM levels were not modulated. Beta-actin was used as loading control. **b** Densitometric analysis of γH2AX expression levels normalised by β-actin (*n* = 3;**P < 0.01;***P < 0.001). **c** Densitometric analysis of NPM expression levels normalised by β-actin (*n* = 3). Uncropped images of blots are shown in Additional file 1: Figure S1b. **d** hCmPCs were treated for 4 h and 8 h with 1 μM Dox, serum-free media were collected and analysed by ELISA assay for NPM expression. NPM was induced at 8h with respect to 4h treatment both in Ctrl- and Dox-treated cells. The difference at 8h between Dox and Ctrl was statistically significant (*n* = 7;*P < 0.05). **e** LDH assay, a marker of cell necrosis, showed that the release does not depend on a passive release from dead cells (*n* = 6). A negative control and positive control were inserted in the assay; the latter is statistically significant vs negative control (*n* = 6; ^*^P < 0.05)

Albeit we did not find ROS or apoptosis increase at these timepoints, since Dox is known to induce DSBs and γH2AX increase, we verified that Dox induced γH2AX after both 4h and 8h of Dox treatment (Fig. 2a, b).

Subsequently, we hypothesised that nucleolar stress could provoke not only NPM translocation to the nucleoplasm, but also its extracellular secretion upon Dox treatment.

First, we assayed intracellular NPM protein expression levels after Dox treatment, and we did not find any changes (Fig. 2a, c).

NPM levels were measured in the supernatant of serum-starved hCmPCs treated with Dox for 4h and 8h. We found that levels of extracellular NPM (eNPM) increased in the supernatants of Dox-treated cells, reaching the statistical significance at 8h compared to control (Fig. 2d). Interestingly, eNPM levels increased with time, i.e. at 8h vs 4h time-point, both in the control and in the Dox-treated cells (Fig. 2d).

To exclude that NPM release was due to necrosis, we measured lactate dehydrogenase (LDH), a marker of cell necrosis, in the conditioned medium; as shown in Fig. 2e, LDH was very low at all time-points and was not modulated by Dox-treatment; thus, necrosis unlikely contributed to NPM release.

Overall, these results indicate that NPM is released in the extracellular space in response to Dox and raise the possibility that eNPM may have a functional role.

### NPM is released in the extracellular space by ultraviolet radiation

**Fig. 3:**
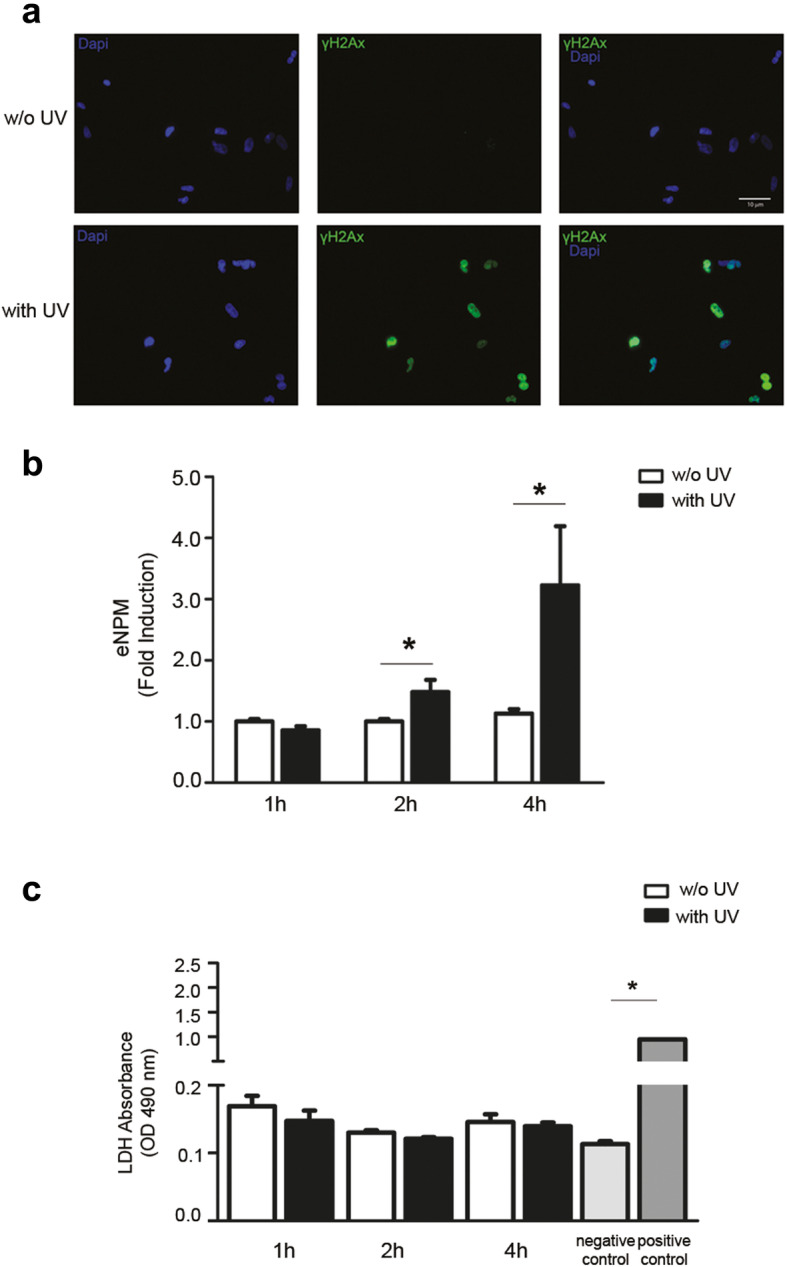
UV-induced DSBs elicit a rapid NPM secretion in the absence of necrosis. **a** hCmPCs were treated with 50mJ/cm^2^ of UV light for 4 h and 8 h (lower panels) or not treated (w/o UV; upper panels) in serum-free media. Representative images of γH2aX (green) immunofluorescence and DAPI (blue)-counterstained nuclei. γH2AX increases upon UV light treatment. Scale bar 10μm. **b** hCmPCs were treated for 1 h, 2 h, and 4h with UV light, and serum-free media were collected and analysed by ELISA assay for the presence of NPM. eNPM was induced at 2h and 4h treatment in UV-treated cells. The difference at 2h and 4h between UV treated and Ctrl was statistically significant (*n* = 9; ^*^P < 0.05). **c** LDH assay, a marker of cell necrosis, showed that the release does not depend on a passive release from dead cells. A negative control and positive control were inserted in the assay, and the latter is statistically significant vs negative control (*n* = 4; ^*^P < 0.05)

In additional experiments, we examined whether ultraviolet radiation (UV), another stimulus known to induce DSBs, provoked NPM secretion in hCmPCs. We confirmed that a 4-h exposure to 50 mJ/cm^2^ UV induced γH2AX compared to control (Fig. 3a). eNPM levels were measured after 1h, 2h, and 4h UV treatment and increased significantly at 2h and 4h compared to untreated cells (Fig. 3b). To exclude that eNPM release was due to necrosis, LDH was measured in the conditioned media and was absent at the tested time-points (Fig. 3c).

These results indicate that NPM is released in the extracellular space also in response to UV suggesting its possible role as a signalling molecule of genotoxic stress.

### NPM levels increase in the plasma of Dox-treated mice

To assess whether NPM secretion could occur in vivo, circulating eNPM was assayed in a mouse model of cardiotoxicity [17, 18]**.**

**Fig. 4:**
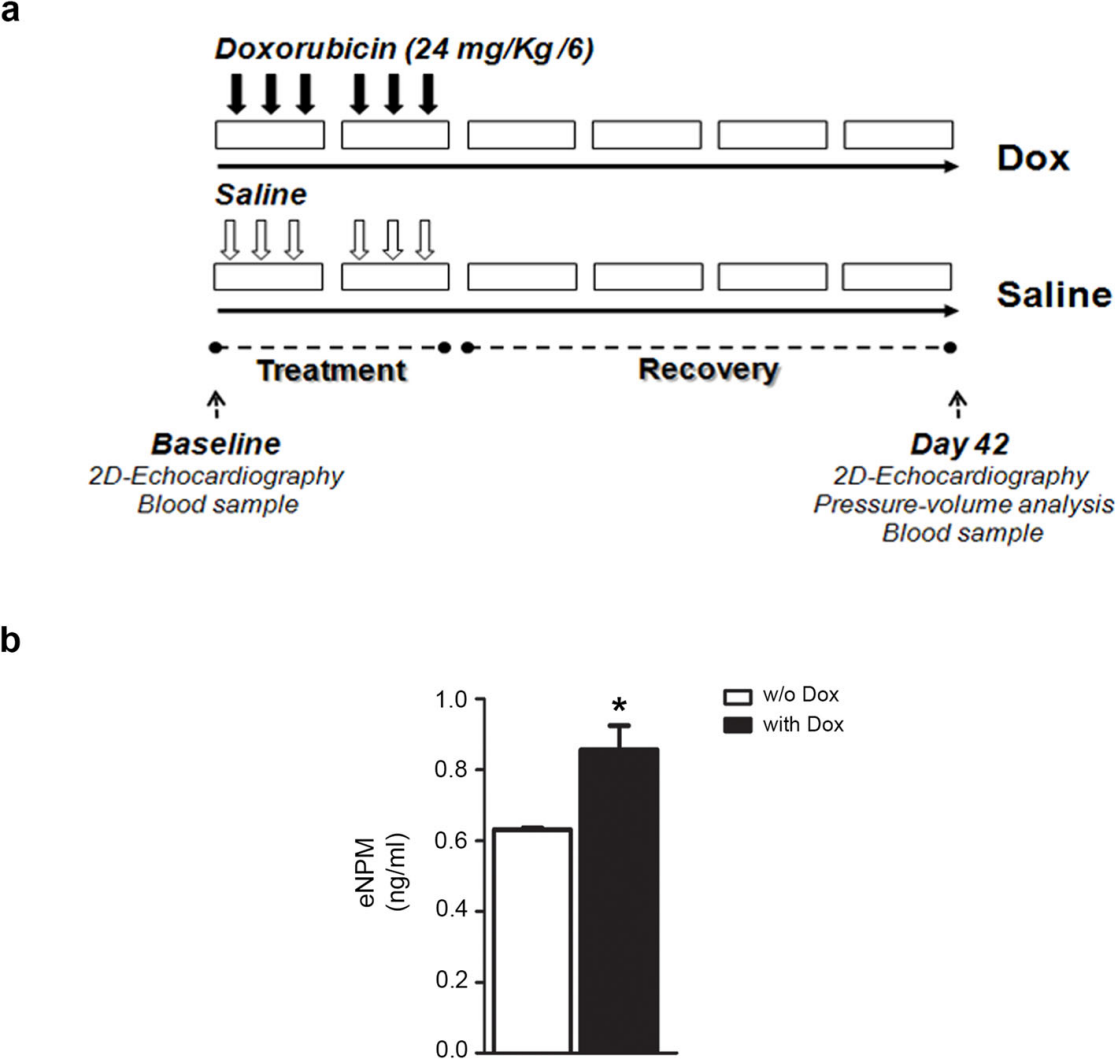
NPM levels increase in plasma of mice treated with Dox. **a** In vivo experimental design. Ten female C57Bl/6 wild-type mice aged 8 to 10 weeks were randomly divided into two groups. In the first group (Dox, *n* = 5), Dox was administered in six equal intraperitoneal injections over a period of 2 weeks. In the second group (saline, *n* = 5), control mice were treated with physiological saline in the same manner as the regimens for the Dox group. Both groups then followed a 4-week recovery period. **b** NPM levels in mice plasma were analysed by ELISA assay at day 42, following 2-week treatment with Dox (black bar) or saline (white bar) and 4 weeks recovery (^*^P <0.05). Five female C57Bl/6 wild-type mice aged 8 to 10 weeks were used for each group

Mice were treated for 2 weeks either with Dox or saline and allowed to recover for 4 weeks for a total of 6 weeks (42 days) (Fig. 4a). Mice treated with Dox exhibited a significant increase of plasma eNPM at day 42 compared to saline-treated mice (Fig. 4b).

These results demonstrate that Dox treatment is associated with an increase of NPM levels in mice plasma.

### Recombinant NPM inhibits cell proliferation

These experiments were aimed at establishing whether eNPM has a biological activity. We treated hCmPCs either with recombinant NPM (rNPM) or its denaurated boiled form (bNPM), as a negative control and evaluated their effects on cell proliferation and cell death after 24h and 48h of treatment.

**Fig. 5:**
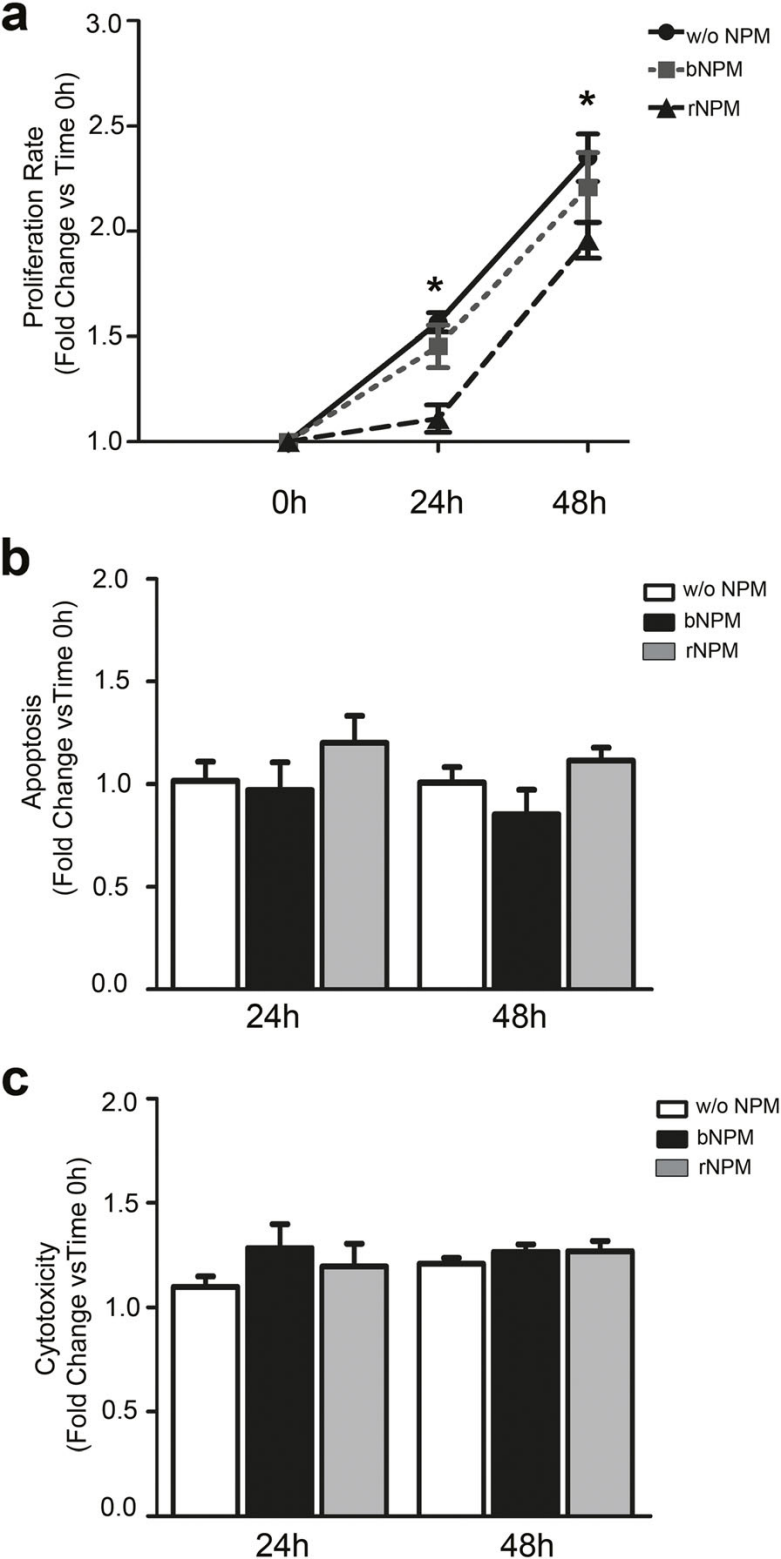
Recombinant NPM decreases proliferation without induction of cell death. hCmPCs were treated either with recombinant rNPM (0.5μg/ml) or boiled NPM (bNPM) for 24h or 48h in full media. Then, proliferation, apoptosis and cytotoxicity assays were performed. **a** Proliferation assay of hCmPCs showed that rNPM significantly decreased the proliferation of hCmPCs at 24h and 48h compared to Ctrl cells (*n* = 7, ^*^P < 0.05). bNPM instead, did not decrease proliferation. **b**, **c** Apotosis and cytotoxicity assays showed no significant differences among the different treatments (*n* = 6)

rNPM significantly decreased hCmPCs proliferation compared to control at 24h and 48h, whereas bNPM had no effect on cell proliferation (Fig. 5a). Neither rNPM nor bNPM significantly increased apoptosis and cytotoxicity at these timepoints (Fig. 5b, c).

Taken together, these results suggest that eNPM is biologically active and decreases proliferation.

### NPM binds to Toll-like receptor 4

In order to investigate the possible mechanism of the autocrine/paracrine effects of eNPM, we explored a potential receptor that may bind NPM and transduce its biological activity. We focused on Toll-like receptor 4 (TLR4), since it has been shown to bind the alarmin High Mobility Group Box 1 (HMGB1), that in human macrophages is secreted together with NPM in response to LPS [9].

**Fig. 6:**
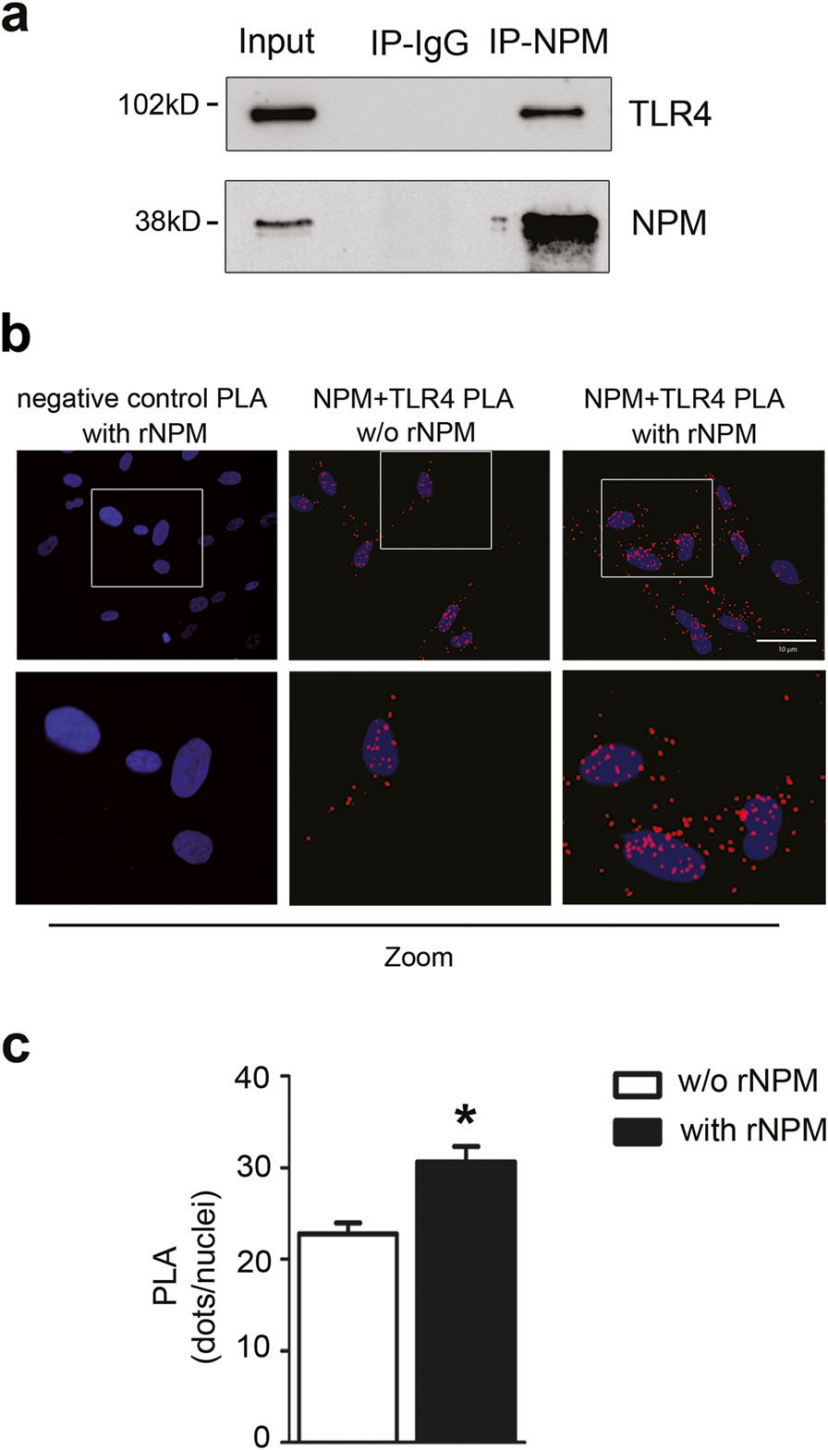
NPM binds to TLR4. **a** Representative image of WB analysis of co-immunoprecipitation between NPM and TLR4 in hCmPCs. Cells were immunoprecipitated with NPM antibody (Ip-NPM) or an irrelevant negative antibody rabbit IgG (IP-IgG). Western blotting with TLR4 revealed that NPM interacts with TLR4 in hCmPCs. The efficiency of immunoprecipitation was assessed with an NPM antibody. One-twentieth of the immunoprecipitated whole-cell extract (Input) was loaded as a reference. Uncropped images of blots are shown in Additional file 1: Figure S1c. **b** Proximity ligation assay of NPM and TLR4 interaction analysis in serum starved hCmPCs treated with rNPM (0.5μg/ml) for 8h. Lower panels represent zoomed in images of the original PLA images. Red dots indicate that even under physiological conditions, NPM interacts with TLR4. When rNPM is administered, the rate of interaction increases. As a negative control, the IgG specific for each antibody (i.e. normal rabbit IgG used as the control of TLR4 antibody) and normal mouse IgG for NPM antibody (left panel) revealed the specificity of NPM and TLR4 interaction, since no red dots were present in the PLA. Scale bar 10μm. **c** Bar graph of the quantification of dots per nuclei (*n* = 5; ^*^P <0.05)

NPM possible interaction with TLR4 was assessed by co-immunoprecipitation. Cell extracts derived from hCmPCs were immunoprecipitated with antibodies to NPM or with immunoglobulin G, as control, followed by Western blotting (WB) to TLR4. Figure 6a showed that TLR4 was clearly detectable in NPM immunoprecipitates, but not in control immunoprecipitates. Moreover, NPM and TLR4 interaction was confirmed in situ by proximity ligation assay (PLA) on serum-starved hCmPCs treated or not with rNPM for 8h. This technique allows to detect whether an extracellular NPM can bind to TLR4. We performed this assay in serum-free media to be sure that NPM did not bind to any component of serum, and we also avoided cell permeabilization step during the assay, to be able to detect a specific interaction between eNPM and the active form of the transmembrane TLR4.

As shown in Fig. 6b, serum deprivation per se induced an interaction between eNPM and TLR4 (central panels). This was probably caused by an extracellular release of endogenous NPM in serum free media (our unpublished results), and the interaction was specific, since we did not detect any red dot in the IgG, used as negative control (Fig. 6b left panels).

In any case, hCmPCs showed an evident increase of TLR4 binding to NPM upon treatment with rNPM (0.5μg/ml), indicated by the increasing number of red dots detectable in the figure (Fig. 6b,c).

To formally demonstrate that the endogenous NPM is secreted extracellularly and is able to bind to TLR4, we performed the experiment described below.

**Fig. 7:**
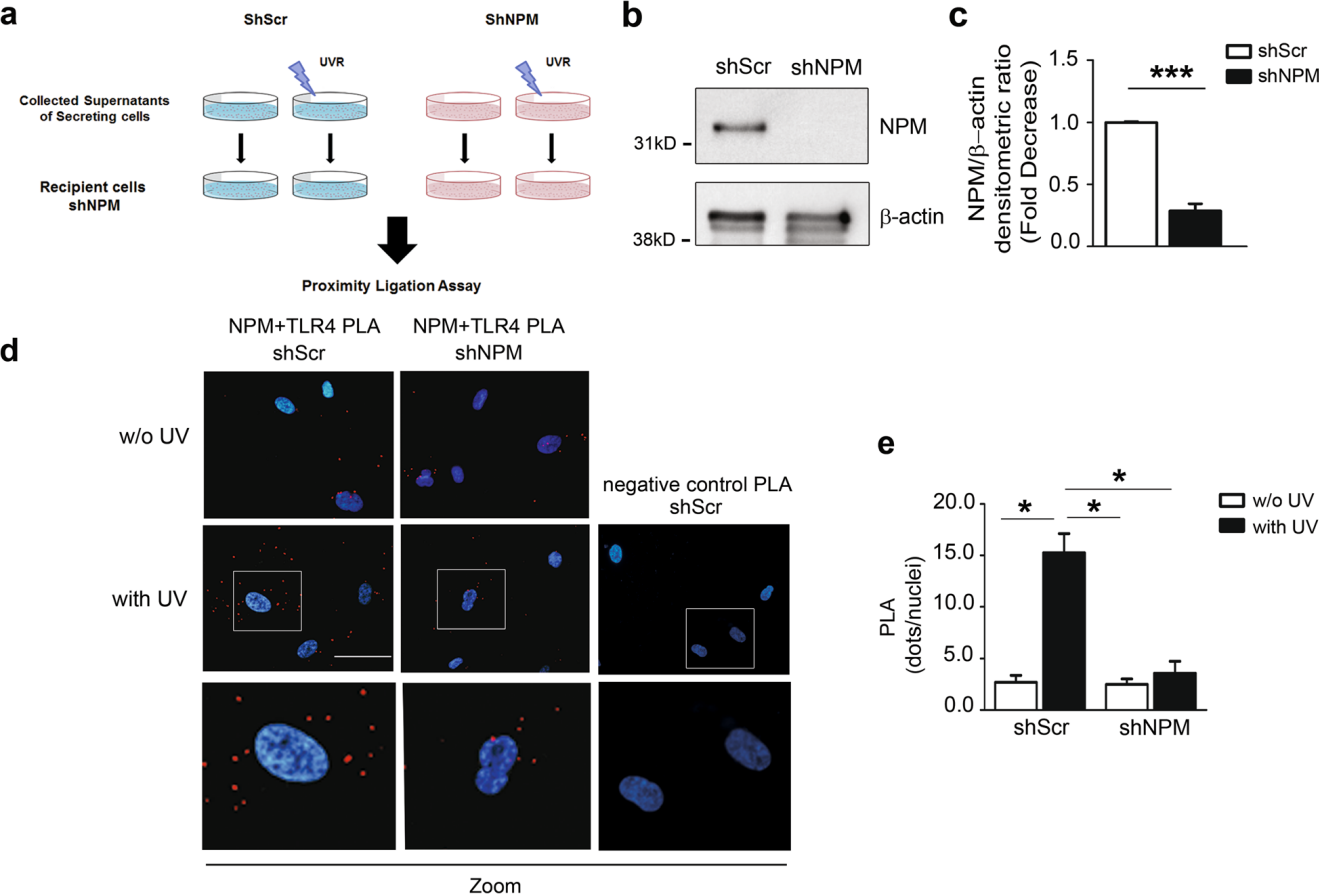
Secreted NPM binds to TLR4 and the binding increases upon UV treatment. **a** Sketch figure of experimental plan. ShScr and ShNPM secreting hCmPCs were treated or not with 50mJ/cm^2^ UV for 4h. The supernatants of secreting cells were then collected and used to culture hCmPCs knocked-down for NPM (shNPM), here referred as recipient cells. **b** Representative WB demonstrating a >70% knockdown of NPM1 expression in hCmPC infected with a lentivirus encoding a NPM1-specific shRNA sequence (ShNPM) compared to control (ShScr). Uncropped images of blots are shown in Additional file 1: Figure S1d. **c** Densitometric analysis of NPM expression levels normalised by β-actin (*n* = 3; ^***^P < 0.001). **d** PLA images of knocked-down NPM cells, cultured in the supernatants of ShScr (left panels) and ShNPM (right panels) treated or not with UV (lower and upper panels, respectively). Lower panels represent zoomed in images of the original PLA images. PLA assay barely detected NPM/TLR4 interaction in recipient cells cultured with the medium derived from untreated ShNPM secreting cells. When recipient cells were kept in the medium of UV irradiated secreting ShScr cells the dots increased (lower left panel). As a negative control, the IgG specific for each antibody (i.e. normal rabbit IgG used as the control of TLR4 antibody) and normal mouse IgG for NPM antibody (right panels) revealed the specificity of NPM and TLR4 interaction, since no red dots were present in the PLA. Scale bar 10μm. **e** Bar graph of the quantification of the PLA of dots per nuclei (*n* = 4; ^*^P < 0.05)

We generated two batches of cells either knocked-down for NPM by RNA interference (ShNPM) or control cells (ShScr), here referred as secreting cells (Fig. 7a). A WB analysis confirmed a >70% decrease of NPM in ShNPM cells (Fig. 7b, c).

Since UV significantly induced NPM secretion, ShScr and ShNPM secreting cells were treated or not with 50mJ/cm^2^ UV for 4h.

The supernatants of secreting cells were then collected and used to culture hCmPCs knocked-down for NPM (shNPM), here referred as recipient cells (Fig. 7a).

This experimental setting reduces the possibility that extracellular NPM could derive from recipient cells and increases the specificity of NPM/TLR4 interaction.

As expected, PLA assay barely detected NPM/TLR4 interaction in recipient cells cultured with the medium derived from untreated ShNPM secreting cells. When recipient cells were kept in the medium of UV irradiated secreting ShNPM cells, the dots slightly increased, but not significantly (Fig. 7d central panels, 7e). NPM/TLR4 interaction was higher when recipient cells were cultured with the supernatant of ShScr secreting cells and reached a maximum when cultured with supernatants of UV-treated ShScr secreting cells (Fig. 7d left panels, 7e). We did not detect any red dots in the IgG, used as a negative control in shScr treated with UV; thus, the interaction was specific (Fig. 7d right panels).

Taken together, these results indicate that eNPM binds to TLR4, suggesting a possible signalling cascade activated by NPM/TLR4 interaction.

### NPM/TLR4 interaction induces nuclear translocation and activation of NFκB

TLR4 belongs to the family of highly conserved proteins TLR and is involved in the activation of the innate immune system, mediating cytokine production via activation of NFκB transcription factor [19].

**Fig. 8:**
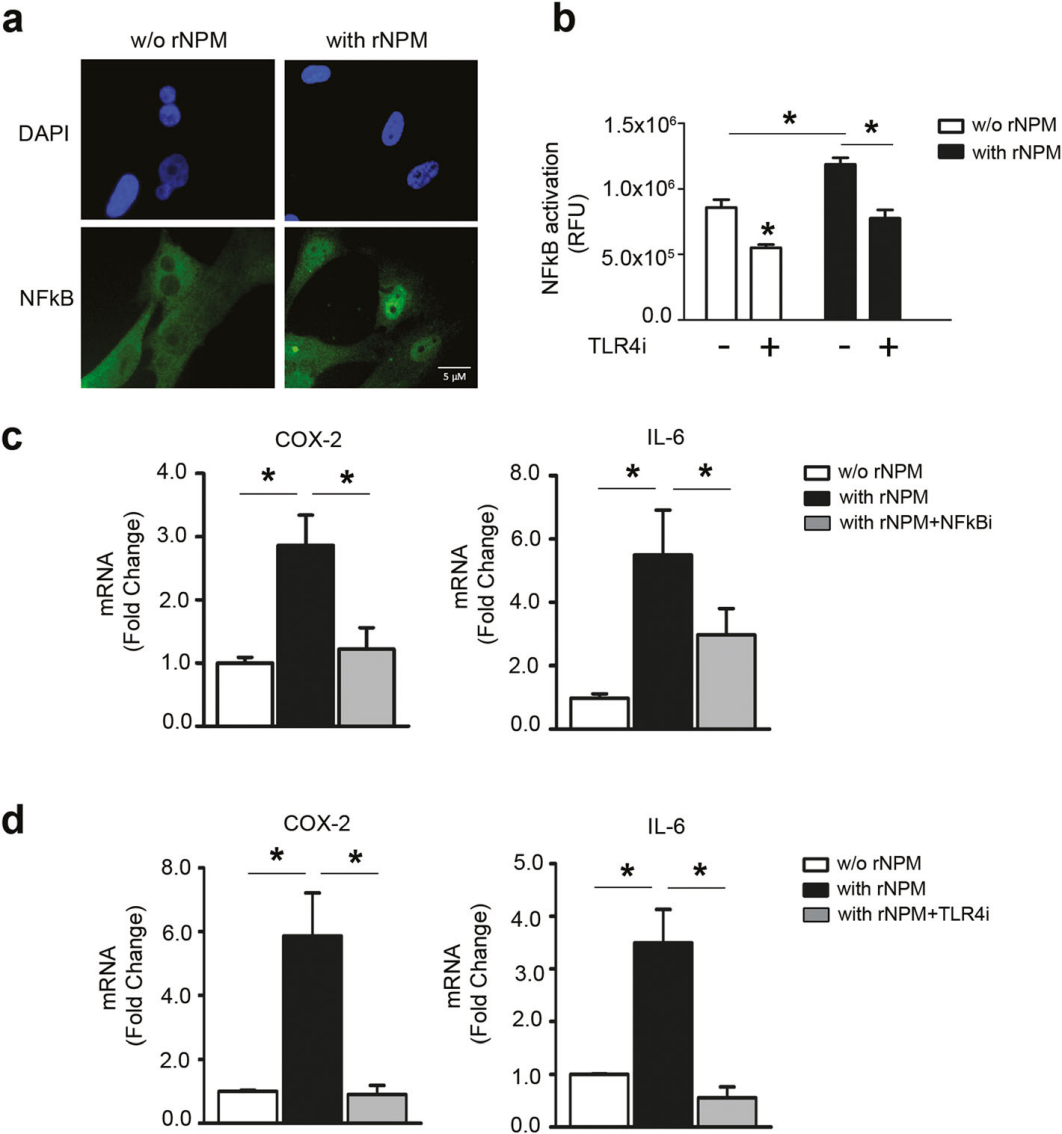
rNPM induces NFκB translocation to the nucleus via TLR4 activation and induces inflammation. **a** Representative image of immunocytochemistry of NFkB (green) with an α-p65rel NFkB antibody, nuclei were counter-stained with DAPI (blue). NFκB translocated in the nucleus in hCmPCs treated for 8h with rNPM (0.5μg/ml) compared to untreated cells (w/o NPM). Scale bar 5μm. **b** hCmPCs were infected for 48h with lentiviral particles bearing the NFkB-GFP reporter gene (Cignal Lenti Reporter Assay, Qiagen). Then cells were pre-treated with 100 μM TLR4i for 30 min and then treated or not for 8h with rNPM (0.5μg/ml). Histogram representing the Cignal Reporter Assay. NFkB activation was assessed by relative fluorescence units (RFU). Fluorescence was increased by rNPM treatment, whereas TLR4i inhibited it (*n* = 6; ^*^P < 0.05). **c** hCmPCs were pre-treated with 5μM NFkB inhibitor (NFkBi) for 30 min and then treated or not for 8h with rNPM (0.5μg/ml). qRT-PCR of COX-2 and IL-6 was significantly reduced by NFkBi upon rNPM treatment (*n* = 6; ^*^P < 0.05). **d** hCmPCs were pre-treated with 100 μM TLR4i for 30 min and then treated or not for 8h with rNPM (0.5μg/ml). qRT-PCR of COX-2 and IL-6 was significantly reduced by TLR4i upon rNPM treatment (*n* = 6; ^*^P < 0.05)

To address whether rNPM induces NFkB nuclear translocation in hCmPCs, an NFκB immunocytochemistry was performed using an anti-p65rel NFkB antibody. Figure 8a showed that upon rNPM treatment NFκB was localised in the nucleus, whereas in untreated cells showed a prevalent cytoplasm localisation.

To investigate TLR4 role in eliciting NPM-induced inflammatory functions, hCmPCs were infected with a NFκB-GFP reporter in the presence or absence of rNPM and of TLR4 inhibitor (TLR4i). As depicted in Fig. 8b, rNPM-induced NFkB pathway activation, that was inhibited by TLR4i.

To assess whether NFκB translocation was followed by transcriptional activation of NFκB target genes, we evaluated Cycloxygenase 2 (COX-2) and interleukin 6 (IL-6) mRNA expression in rNPM treated vs control. rNPM treatment elicited both COX-2 and IL-6 mRNA expression increase, that was significantly decreased in the presence of an NFκB inhibitor, confirming NFκB transcriptional activity induction (Fig. 8c).

Moreover, to investigate the involvement of TLR4 in NPM capacity to induce the expression of NFκB target genes, we repeated the same experiments using a TLR4 inhibitor. As indicated in Figure 8d, both IL-6 and COX-2 induction by rNPM were significantly decreased by TLR4 inhibition.

These findings indicate that NPM/TLR4 interaction is functional and activates NFκB-inflammatory signal transduction pathway.

### TRL4 regulates NPM secretion in response to Dox

TLR4 is a well-known LPS receptor [20], and it has been previously shown that upon TLR4 activation with LPS, both human macrophages and endothelial cells secrete concomitantly the alarmin HMGB1 and NPM [9]. TLR4 was also shown to be involved in Dox-induced cardiotoxicity [21, 22], and Dox was shown to induce TLR4 expression in human macrophages, mediating systemic inflammation in cancer patients [23, 24].

**Fig. 9:**
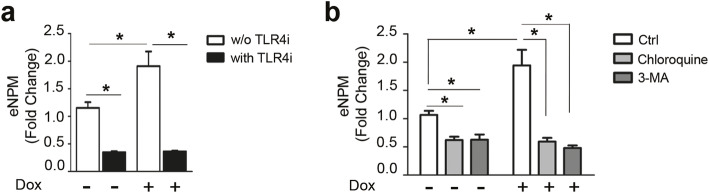
TRL4 and an autophagy-based mechanism regulate NPM secretion in response to Dox. **a** hCmPCs were pre-treated with 100 μM TLR4i for 30 min. Then 1 μM Dox was added to cells for further 8 h. Afterwards, serum-free media were collected and analysed by ELISA assay for the presence of eNPM. As already observed, Dox induced a significant secretion of NPM vs Ctrl while the inhibition of TLR4 inhibited NPM secretion in the extracellular milieu (*n* = 4; ^*^P < 0.05). **b** hCmPCs were pre-treated with two autophagy inhibitors, 50 μM chloroquine (Chl) and 5μM 3-MA (5) for 30 min, then 1 μM Dox was added to cells for further 8 h. Serum-free media were collected and analysed by ELISA assay for the presence of eNPM. Dox induced a significant secretion of NPM vs Ctrl while the treatment with Chl and 3-MA significantly inhibited NPM secretion in the extracellular milieu (*n* = 7; ^*^P < 0.05)

We hypothesised that TLR4 activation could induce NPM secretion. To evaluate this possibility, hCmPCs were exposed for 8h to Dox with or without TLR4i and eNPM levels were measured in the supernatants. We found that TLR4i significantly inhibited NPM secretion, both under control conditions and upon Dox exposure (Fig. 9a). These findings show that TLR4 modulates NPM secretion and suggest the existence of an autoregulatory-loop between NPM and TLR4 in response to Dox.

**Fig. 10:**
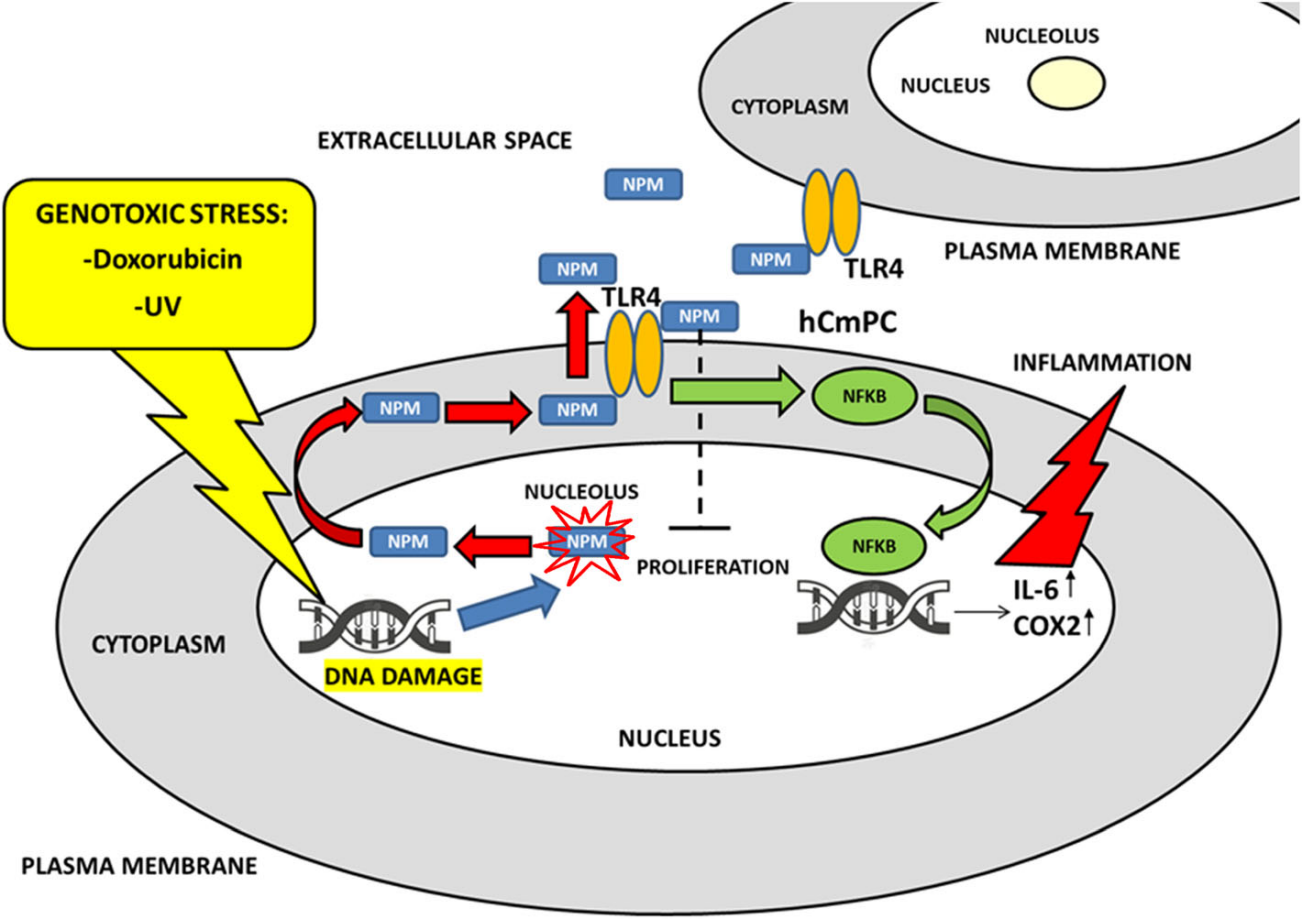
Proposed mechanism of genotoxic stress-mediated NPM release and extracellular NPM functions on hCmPCs. Following DSB formation caused either by Dox-induced nucleolar stress or UV light treatment, the nucleolus is rapidly disrupted and NPM is released from the nucleolus to the nucleoplasm, subsequently to the cytoplasm and then is rapidly secreted in the extracellular space by an active mechanism, that requires TLR4 activity and an autophagy mechanism. Our results show that NPM binds to TLR4 and activates a TLR4-dependent signalling cascade that causes NFkB translocation in the nucleus and the transcription of IL-6 and COX-2, thus causing inflammation. The extracellular NPM is also able to inhibit cell proliferation without induction of cell death. In conclusion, following genotoxic stress, NPM acts similarly to an alarmin in hCmPCs, being rapidly secreted and promoting cell cycle arrest and TLR4/NFκB-dependent inflammatory response

Moreover, recently HMGB1 has been shown to be secreted extracellularly by an autophagy unconventional mechanism in psoriasis by keratinocytes [25].

We wanted to determine whether a similar active mechanism could be involved in NPM extracellular release. To this aim, we took advantage of two autophagy inhibitors, i.e. chloroquine, that is a pharmacological inhibitor of autophagy and also of TLR4, and 3-methyladenine (3-MA), a selective autophagy inhibitor. We found that both the autophagy inhibitors significantly inhibited NPM extracellular release upon Dox treatment (Fig. 9b).

Therefore, an autophagy-based secretion mechanism seems to be involved in NPM extracellular release in hCmPCs.

## Discussion

Herein, we describe a novel role of the nucleolar protein NPM in the early response to stress of hCmPCs. We found that in hCmPCs, following Dox treatment the structure of the nucleolus was rapidly affected, NPM was delocalised from the nucleolus to the nucleoplasm, and RNA polI was inhibited. Two different stimuli were used in order to induce nucleolar stress: UV light, that was reported to induce NPM delocalisation in keratinocytes [26] and in mouse embryonic fibroblasts [27], and Dox, an anthracycline that we previously showed to induce NPM shuttling from the nucleolus to the nucleoplasm in NRCMs and mCPC [12]. We showed that both stimuli induce NPM release of the protein in the extracellular milieu that occurs in the absence of apoptosis, cytotoxicity and ROS increase, supporting the idea that NPM secretion could represent a critical and precocious step during genotoxic stress response. Indeed, previous studies have shown that NPM can be found in the extracellular space in response to different stress stimuli [9, 10, 28, 29].

Under these treatment conditions, we did not find any difference in endogenous NPM protein expression, most probably because of the low quantity of NPM that is released in the supernatants (in the range of ng/ml), compared to the internal amount of NPM which is instead, highly expressed. Probably, the WB technique is not so sensitive in the detection of very small quantity changes. Another possible explanation is that NPM expression is increased in response to genotoxic stress to balance the little amount of the secreted protein, both hypotheses need further investigations.

Interestingly, we found that in a mouse model of Dox-induced cardiotoxicity circulating eNPM was increased in plasma compared to control mice.

We observed that in hCmPC, both Dox and UV were associated to an induction of γH2AX, a marker of DSBs. Several works reported DSBs as an upstream event of the DNA damage response (DDR)-dependent secretory signature that can function in a paracrine manner through the release of not yet identified signalling molecules that lead to senescence or apoptosis of the surrounding cells [30, 31].

Our results indicate that rNPM inhibits hCmPCs proliferation in absence of apoptosis and cytotoxicity and behaves as an active molecule that binds to TLR4. We demonstrated NPM and TLR4 interaction by both immunoprecipitation assay and PLA, a technique that allows to determine the extracellular binding of NPM. The latter was performed employing both the exogenous rNPM, and the endogenous NPM released upon UV exposure. Our results showed that serum deprivation per se induces NPM release in small quantity, and the basal interaction of NPM and TLR4 is further upregulated in the presence of UV. To demonstrate that the endogenous NPM is released extracellularly and binds to TLR4, hCmPCs were treated or not with UV to induce endogenous NPM release in the supernatants, that were then collected and used to culture a second batch of hCmPC silenced for NPM, used as recipient cells, in order to avoid the release of endogenous NPM from these cells. We then performed PLA assay to demonstrate that UV-secreted NPM was able to specifically bind to TLR4 receptor. As expected, NPM/TLR4 interaction in recipient cells cultured with the medium derived from untreated NPM knocked-down secreting cells were very low and these results did not change when recipient cells were kept in the medium of UV-irradiated NPM knocked-down cells, thus demonstrating the specificity of TLR4/NPM interaction. NPM/TLR4 interaction was higher when recipient cells were cultured with the supernatant of control (shScr) secreting cells and reached a maximum with supernatants of UV-treated control secreting cells. Overall, these results demonstrate that the endogenous NPM is secreted extracellularly and is able to bind to TLR4 exogenously. The PLA technique was chosen for its sensitivity and to demonstrate that this interaction was extracellular, since we did not permeabilize the cells.

Further, we showed that NPM activates TLR4 signalling cascade by inducing NFkB translocation in the nucleus and the transcriptional activation of its target genes COX-2 and IL-6. Moreover, IL-6 and COX-2 transcriptional induction was decreased by both NFkB inhibitor and TLR4 inhibitor. These data demonstrate that extracellular NPM induces inflammation via a TLR4/NFkB mechanism.

These findings are in agreement with previous studies that showed that rNPM induced inflammation in macrophages, causing the release of cytokines (such as, TNF-α, IL-6, and MCP-1) [9]. Further, rNPM induced endothelial expression of intercellular adhesion molecule 1 (ICAM-1), a molecule associated with proinflammatory pathways [9]. Moreover, our data are in agreement with a recent paper that showed an interaction of NPM1 with myeloid differentiation protein-2 (MD-2), a protein that associates with TLR4, in human leukemia monocytic cell line THP-1 [32].

Although NFkB activity is known to induce proliferation in a cancer cell, it has been demonstrated that in normal mouse fibroblasts and primary keratinocytes it behaves inhibiting proliferation [33]. This occurs through an E2F-dependent gene expression inhibition caused by the disruption of the interaction between activator E2Fs (E2F1, E2F2, and E2F3) and the HAT cofactor transactivation/transformation-domain-associated protein [33].

Our results are in agreement with this paper’s results, since we found that rNPM decreases proliferation and activates the NFkB pathway in hCmPCs.

Interestingly, TLR4 is a receptor capable to recognise molecules and few proteins, among which HMGB1, an alarmin that is secreted following necrosis via TLR4 stimulation [34].

Recently, HMGB1 has been shown to be secreted extracellularly by an autophagy-based unconventional mechanism in psoriasis by keratinocytes [25].

We found that an autophagy-based secretion mechanism seems to be involved in NPM extracellular release in hCmPCs, since two different autophagy inhibitors decrease NPM extracellular release upon Dox treatment.

Herein, we found that NPM is a novel ligand of TLR4, that activates an inflammatory signalling cascade, that extracellular NPM inhibits proliferation and that TLR4 and autophagy-based secretion mechanisms are involved in NPM secretion, since their inhibition strongly impairs NPM secretion following Dox treatment (Fig. 10).

## Conclusions

Our study suggests a possible role of NPM in cardiac inflammation. Under our experimental conditions, NPM behaved similarly to an alarmin linking nucleolar stress to TLR4 stimulation, which may represent a novel molecular mechanism of genotoxic stress-induced cardiotoxicity. Although further investigations are needed, we suggest that eNPM blockade may represent a novel strategy to attenuate anthracycline-induced cardiotoxicity and that eNPM may represent a new early marker of anthracycline-induced cardiac damage.

## Methods

### Cell culture

Human cardiac mesenchymal progenitor cells (hCmPC) were isolated from the right auricle of patients undergoing cardiac surgery at the Centro Cardiologico Monzino-IRCCS. hCmPCs were mycoplasma-free and obtained adapting two different methods previously described [35, 36]. An additional overnight (O/N) step with 0.3 mg/mL collagenase NB4 (Serva, Germany) was performed to improve hCmPC recovery efficiency.

### Drug treatments

Cells were treated with the following drugs administered in a serum-free medium (Ham’s F-12). Doxorubicin (Sigma, St. Louis, MO, USA) was dissolved in DMSO and used 1μM, and recombinant NPM (rNPM) was purchased from Abcam (Ab114194) and used 0.5 μg/ml. TLR4 inhibitor (1-methylethyl 2-(acetylamino)-2-deoxy-α-d-glucopyranoside 3,4,6-triacetate, C34 5373/10 Tocris Bioscience) was dissolved in water and used 100μM. NFkB activation inhibitor (6-amino-4-(4-phenoxyphenylethylamino) quinazoline, Millipore Corp 481406) was dissolved in DMSO used at a final concentration of 5μM, chloroquine (Sigma, St.Louis, MO, USA) was dissolved in water and used at a final concentration of 50 μM and 3-methyladenine (3-MA) (Sigma, St. Louis, MO, USA) was dissolved in DMSO and used at a final concentration of 5mM.

### Lentiviral infection

Lentiviral supernatants were produced using standard procedures. hCmPCs were infected for 2 hours (h) with lentiviral supernatants and allowed to recover in a complete fresh medium for additional 24h. Afterwards, puromycin-containing medium (0.5μg/ml, Sigma) was added to the cells. MISSION shRNA-lentiviral control and NPM1-specific (NM_002520; TRCN0000062270) constructs were purchased from Sigma.

### UV treatment

Subconfluent hCmPCs were challenged with 50 mJ/m^2^UV (UV Stratalinker 1800 STRATAGENE). Serum-free media were added to cells, and after 4h, supernatants were collected, filtered and immediately froze at –80°C for NPM quantification.

### Immunocytochemistry

Subconfluent hCmPCs grown on coverslips were treated with either Dox or rNPM. Cells were fixed 30 min with 4% paraformaldehyde in PBS and permeabilized 5 min with 0.1% Triton X-100. The following primary antibodies diluted in 1% BSA were incubated 1h at 25°C: NPM1 (1:400 Abcam ab86712), γH2AX (1:400; 05-636Millipore), α-TLR4 (1:100 in PBS; Santa Cruz), and α-p65rel NFkB (1:200; F6 Santa Cruz). After PBS washes, secondary antibodies were added 1h at R.T.: goat α-mouse IgG-FITC and goat α-rabbit IgG-Texas Red (1:200 in PBS; Jackson Immunoresearch Laboratories, West Grove, PA, USA). Nuclei were stained with DAPI (1:10,000 in PBS; Sigma). Images were acquired with the ApoTome System (Zeiss) connected with an Axiovert200 inverted microscope (Zeiss); image analysis was performed with ZEN software (Zeiss).

### Immunoprecipitation

Immunoprecipitations were performed as previously described [37]. Human CmPCs were resuspended in lysis buffer containing 50 mM HEPES (pH 7.5), 250 mM NaCl, 1mM EDTA, 0.5 mM EGTA, 1 mM DTT, 0.1% Tween 20, 10% glycerol, 1 mM phenylmethylsulfonyl fluoride (PMSF), 10 mM Na_3_VO_4_, 50 mM NaF, and protease inhibitors (complete EDTA-free protease inhibitor mixture tablets; Roche Applied Sciences). Immunoprecipitations were performed overnight at 4C with protein A/G agarose and 5 μg of NPM antibody (ab245326, Abcam). Immune complexes were resuspended in 2x Laemmli buffer, separated by SDS-polyacrylamide gel electrophoresis (PAGE), and immunoblotted with TLR4 antibody (35; Santa Cruz) and NPM antibody (ab15440; Abcam).

### In situ proximity ligation assay (PLA)

The in situ proximity ligation assay (PLA) [38] with the DuoLink in situ detection kit (Sigma) was used to detect and quantify the interaction of NPM with TLR4. Cells were grown on slides in 35 mm plates, fixed with 4% paraformaldehyde solution for 10 min, rinsed with PBS, and incubated 1h in the blocking buffer. α-NPM1 (ab86712) 2.5μg/ml or α-TLR4 (sc-10741) 2μg/ml, α-rabbit PLUS/MINUS, and α-mouse PLUS/MINUS PLA probes were incubated 1h at 37°C and then washed. Hybridization, ligation and amplification steps were performed for 30 min and polymerase reaction for 120 min at 37°C. Nuclei were counter-stained with DAPI (1:10,000 in PBS; Sigma). Images were captured with the ApoTome System (Zeiss) as described above. hCmPCs were seeded on rounded glass coverslips coated with porcine gelatin (2%; Sigma-Aldrich). For PLA on NPM1 knocked-down cells, coverslips were removed from the cell culture plate and placed in conditioned media of cells treated or not with UV for 4h. Subsequently, coverslips were removed and processed for PLA.

### NFKB nuclear translocation assay

NFKB nuclear translocation assay upon NPM treatment was assessed by NFκB-GFP gene, i.e. a GFP gene under the control of a minimal (m) CMV promoter bearing tandem repeats of the NFκB transcriptional response element. hCmPCs were infected for 48h with lentiviral particles bearing the NFkB-GFP reporter at a multiplicity of infection (MOI) of 1TU/cell, as indicated by manufacturers’ (Cignal Lenti Reporter Assay, Qiagen). Then, cells were either treated or not-treated with rNPM (0.5μg/ml) for 8h. The GFP signal was detected by the Perkin Elmer EnSight microplate reader.

### Flow cytometry

Subconfluent hCmPCs were treated 8h with Dox. Afterwards, cells were treated 30 min with 1 μM dichlorofluorescein (DCF; H_2_DCFDA, Life Technologies) for ROS detection, according to the manufacturers’ instructions.

Apoptosis was detected by APC Annexin-V (APC; Annexin V; BD Pharmingen) staining according to manufacturers’ instructions. Briefly, cells were treated with Dox for 8h, 24h, 48h, and 72 h and incubated with Annexin-V-APC (0.25 mg/ml) for 15 min at room temperature in the dark. The stained cells were then analysed by flow cytometry within 30 min from staining. 50x10^3^ cells for each treatment were collected and analysed with the MACS Quant® Analyzer cytometer (Miltenyi Biotec GmbH).

### Necrosis quantification

Cells were seeded (4000 cells/well) in a 96-well. After 24h, the cells were treated with either Dox or UV. Necrosis was assessed by LDH assay (CytoTox96® Non-Radioactive Cytotoxicity Assay, Promega). hCmPC supernatants were read at 490nm with the Victor3 1420 Multilabel Counter microplate reader (Perkin Elmer, Waltham, MA, USA).

Bovine heart LDH furnished with the cytotoxicity assay was used as LDH-positive control. No-cell control, i.e. wells without cells, was used as LDH-negative control to determine culture medium background.

### Proliferation and cytotoxicity assays

hCmPCs were seeded in a 96-well plate culture (4000 cells/well) and incubated at 37°C O/N. hCmPCs were treated with either recombinant rNPM (0.5μg/ml) or boiled NPM (bNPM) (i.e. rNPM boiled at 100°C for 15 min). Afterwards, media were removed and changed with serum-starved media for additional 40h. For proliferation assay, plates were frozen at −80°C for further 48h, for apoptosis assay was immediately processed.

#### Proliferation assay

CyQuant cell proliferation assay kit (C7026, Invitrogen) was performed on thawed plates. Detection reagent was added to the wells for 1h at 37**°**C. Fluorescence was measured at 508nm Ex/527nm Em on Victor 3 1420 Multilabel Counter (Perkin Elmer, Waltham, MA, USA).

#### Cytotoxicity assay

CellTox™ Green Cytotoxicity Assay (Promega) was used to test cell death. The fluorescence was measured at 500nm Ex/530nm Em at Victor 31420 Multilabel Counter (Perkin Elmer).

#### Apoptosis

Caspase-Glo® Reagent (Promega G8090) was added on cells, causing their lysis followed by caspase cleavage of the substrate. The liberated free aminoluciferin, which is consumed by the luciferase, generates a “glow-type” luminescent signal that is proportional to caspase-3/7 activity. The luminescent signal was read on EnSight microplate reader (Perkin Elmer).

### Western blot

Cells were lysed in 100 mM Tris (pH 6.8), 20% glycerol, and 4% SDS buffer. Protein concentrations were determined by BCA kit (Pierce, Rockford, IL, USA), then 200 mM dithiothreitol was added boiled 5 min. Proteins were separated by SDS-PAGE and transferred to the nitrocellulose membrane. Membranes were blocked with 5% nonfat dry milk powder in 0.05% Tween20 phosphate-buffered saline (PBS-T) for 1h. Primary antibodies were incubated 2h at RT or O/N at 4°C. Membranes were washed with PBS-T and incubated 1h with secondary antibody conjugated with horseradish peroxidase. After washes blots were developed with Amersham-ECL-Plus and exposed to ChemiDoc (Bio-Rad, Hercules, CA, USA). Protein levels were evaluated by densitometric analysis using Image Lab Software (Bio-Rad). The following primary antibodies were used: α-NPM1 (ab86712; Abcam), α-βactin peroxidase (clone AC-15; Sigma A3854), and α-γ2HAx (1:400; Millipore 05-636).

### ELISA assay

15x10^3^ cells were seeded on a 6-well plate. After 24h of treatment with Dox, supernatants were collected and passed through 0.45μm filters and froze at −80°C. 100μl of supernatants or 50μl of mice plasma was used for NPM-ELISA (DBA Italia, SEC664Hu). NPM levels were measured by absorbance at 450nm by Victor3 1420 Multilabel Counter microplate reader (Perkin Elmer).

### Quantitative real-time PCR analysis

Total RNA was extracted using QIAzol (Qiagen). cDNA was generated by the SuperScript First-Strand Synthesis System (Invitrogen), and real-time PCR was performed with the SYBR-GREEN RT-qPCR method (Qiagen) using QuantStudio5 Realtime-PCR. mRNA expression was normalised to 18S rRNA. Relative expression was calculated using the comparative Ct method (2^–ΔΔCt^). The following primers were used for RT-qPCR:

COX-2

Forward 5′CTTCACGCATCAGTTTTTCAAG-3′

Reverse 5′TCACCGTAAATATGATTTAAGTCCAC-3′

IL-6

Forward 5′GATGAGTACAAAAGTCCTGATCCA-3′

Reverse 5′CTGCAGCCACTGGTTCTGT-3′

18S

Forward 5′CGAGCCGCCTGGATACC-3′

Reverse 5′CATGGCCTCAGTTCCGAAAA-3′

45S

Forward 5′GAACGGTGGTGTGTCGTTC-3′

Reverse 5′GCGTCTCGTCTCGTCTCACT-3′

### Animals and experimental protocol

The mouse model of Dox-induced cardiotoxicity was developed by the Centro Cardiologico Monzino as previously described [17]. Briefly, 10 female C57Bl/6 wild-type mice (Charles River Laboratories) aged 8 to 10 weeks were randomly divided into two groups. In the first group, Dox was administered in six equal intraperitoneal injections over a period of 2 weeks (*n* = 5; 4mg/kg each; cumulative dose, 24mg/kg). Control mice (*n* = 5) were treated with physiological saline in the same manner as the Dox-group, then mice were let recover for 4 weeks for a total of 6 weeks (42 days). At day 42, mice were sacrificed and plasma collected for NPM concentration. No blinding to the group was assigned.

### Statistical analysis

All data are expressed as means ± standard error (SEM) from at least 3 independent experiments. Because of the novelty of the study, whose primary objectives are mainly descriptive and exploratory in nature, the minimum sample size has not been predetermined. Each variable was checked for normality distribution by the D’Agostino and Pearson omnibus normality test. The difference between the two groups was compared either by the two-tailed Mann-Whitney or Wilcoxon rank test for nonparametric groups or by two-tailed Student t test for parametric variables using GraphPad Prism software (Version 5.0). *P* < 0.05 was considered statistically significant.

## Supplementary Information

**Additional file 1: Figure S1.** Representative FACS for Annexin V and uncropped Western blots. a) Representative image of Annexin V APC FACS measurements of hCmPCs treated or not with 1μM Dox for 8h 24h, 48h and 72h corresponding to Fig. 1e. b) Uncropped Western blot images corresponding to Fig. 2a. c) Uncropped Western blot images corresponding to Fig. 6a. d) Uncropped Western blot images corresponding to Fig. 7b.

**Additional file 2.** Raw data relative to all figure graphs.

## Data Availability

All data generated during this study are included in this published article. Raw data are available as an Additional file 2.
