# Supplementary figures and images for: Doxorubicin induces an alarmin-like TLR4-dependent autocrine/paracrine action of Nucleophosmin in human cardiac mesenchymal progenitor cells
Source: BMC Biol. 2021 Jun 16;19:124. doi: 10.1186/s12915-021-01058-5 (PMC8210386; doi:10.1186/s12915-021-01058-5)

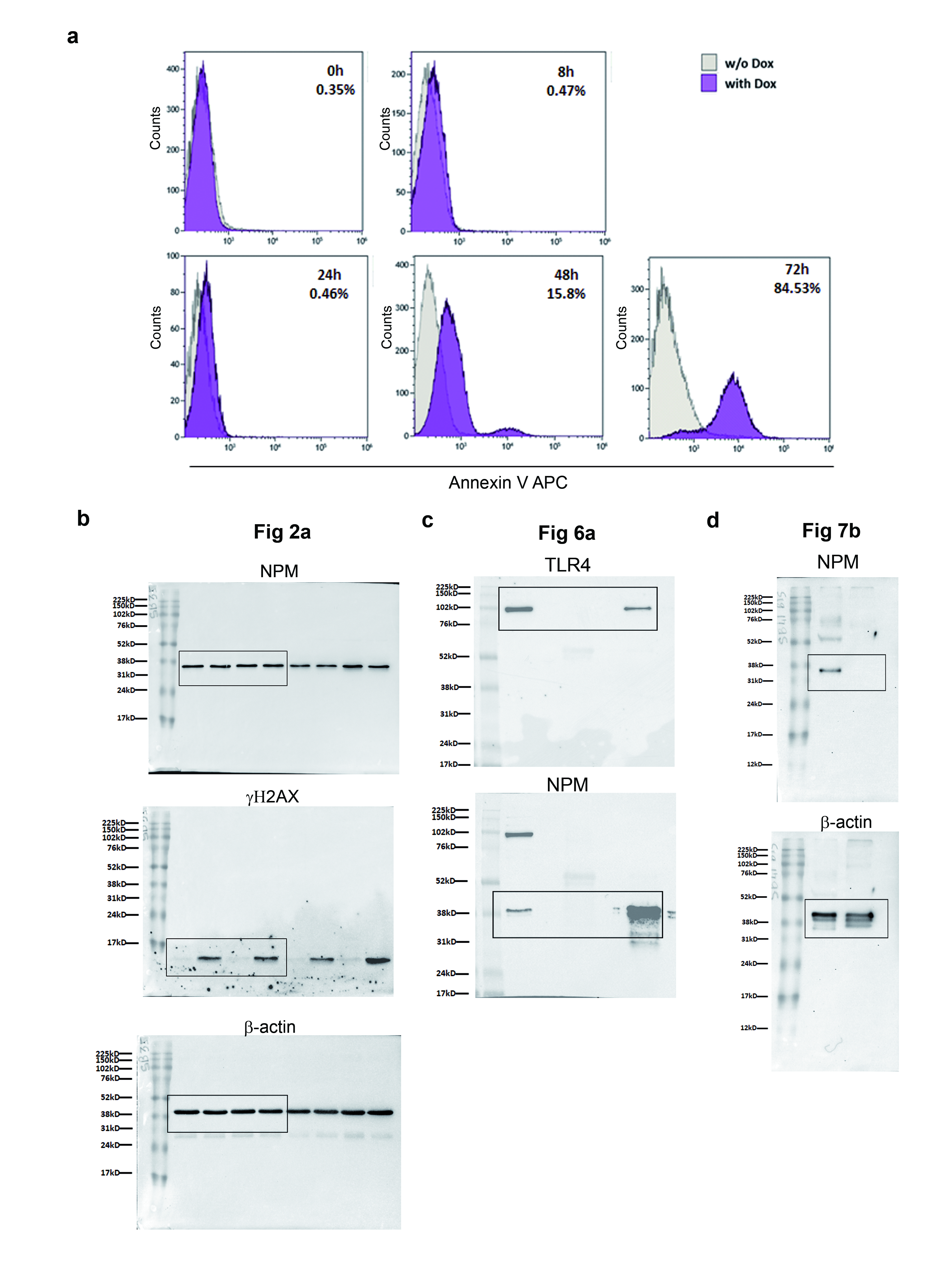

Supplement: Supplementary file 1 — Additional file 1: Figure S1. Representative FACS for Annexin V and uncropped Western blots. a) Representative image of Annexin V APC FACS measurements of hCmPCs treated or not with 1μM Dox for 8h 24h, 48h and 72h corresponding to Fig. 1e. b) Uncropped Western blot images corresponding to Fig. 2a. c) Uncropped Western blot images corresponding to Fig. 6a. d) Uncropped Western blot images corresponding to Fig. 7b. [file 12915_2021_1058_MOESM1_ESM.tif]
